# Supplementary material for: A Putative Effector Pst-18220, from Puccinia striiformis f. sp. tritici, Participates in Rust Pathogenicity and Plant Defense Suppression
Source: Biomolecules. 2024 Aug 31;14(9):1092. doi: 10.3390/biom14091092 (PMC11430752; doi:10.3390/biom14091092)
Supplement: Supplementary file 1 [file biomolecules-14-01092-s001.zip › biomolecules-3135860-supplementary.pdf]

Table S1. The primers used in this study.

Name	Sequences (5′-3′)	Purpose
18220-ORF-BamHI-F	CGGGATCCATGTTGTTGCCCGTCATCTTG	PHB-18220
18220-ORF-XbaI-R	GCTCTAGATCACTTCTTCGAGCCACGAA	construction
18220$^{\Delta SP}$-BamHI-F	CGGGATCCATGAGTCCGCTTTCGATTCC	PHB-18220$^{\Delta SP}$
18220$^{\Delta SP}$-XbaI-R	GCTCTAGATCACTTCTTCGAGCCACG	construction
PstEF1-qRT-F	TTCGCCGTCCGTGATATGAGACAA	*Pst* reference gene
PstEF1-qRT-R	ATGCGTATCATGGTGGTGGAGTGA	
18220-qRT-F	GTTGTTGCCCGTCATCTTG	qRT-PCR
18220-qRT-R	CCACCTCTGAATGCTCTCTTT	
18220VIGS1-F	ATATTAATTAAATATCCATTCTGCTGGTCTACC	
18220VIGS1-R	TATGCGGCCGCGCTCTCTCTTCATCAGGGCTCT	vector construction
18220VIGS2-F	ATATTAATTAATGGGAGAAAGTGTGTCAAGGA	for HIGS
18220VIGS2-R	TATGCGGCCGCGGCTTGAAGTCGGGATTGTA	
AtActin-F	TCTTTCTCTTTATGCCAGTGGT	*Arabidopsis* reference
AtActin-R	TGCATACCCCTCATAGATAGGA	gene
AtPCRK1-F	CATACCTGCATGAAGAAATGG	qRT-PCR
AtPCRK1-R	GATGTGAGACGGCCGGTTTG	
AtPCRK2-F	GATGTTTCTGGAACGAGCAC	qRT-PCR
AtPCRK2-R	AACCCTCTTTTGCCGAGCTG	
AtBIK1-F	GAACATCTTACTTGATGCGGAC	qRT-PCR
AtBIK1-R	AAGCAAAACTCCGAAACTGTAC	

Table S2. The protein information used in phylogenetic tree.

Genbank accession No.	Protein name	Species
XP_047807437.1	Pst134EA_011604	*Puccinia striiformis* f. sp. *tritici* (*Pst*)
KAI9609034.1	KEM48_003117	*Pst* (PST-130)
POW19652.1	PSHT_04414	*Pst*
XP_047807438.1	Pst134EA_011605	*Pst*
POW06476.1	PSTT_08968	*Pst*
KAI7958196.	MJO29_006413	*Pst*
KAI7955244.1	MJO28_005644	*Pst*
XP_047808702.1	Pst134EA_033381	*Pst*
KAI7938097.1	MJO28_015017	*Pst*
KAH9442138.1	Pst134EB_028402	*Pst*
POW14410.1	PSHT_07440	*Puccinia striiformis*
KNE97685.1	PSTG_09089	*Pst* (PST-78)
POW19992.1	PSHT_04131	*Puccinia striiformis*
KAA1093288.1	PGTUg99_012661	*Puccinia graminis* f. sp. *tritici*
KAI9599948.1	KEM48_000059	*Pst* (PST-130)
PP048920	Pst-18220	*Pst* (CYR32)
